# Novel *WAC* gene variant identified in the first documented case of DeSanto-Shinawi Syndrome in India

**DOI:** 10.1186/s40348-025-00193-1

**Published:** 2025-05-10

**Authors:** Aradhana Dwivedi, Lakshita Chauhan, Pramod Kumar, Aashna Nanda, V. Y. Jayakrishnan

**Affiliations:** 1 Division of Clinical Genetics, Advance Centre of Pediatrics Medicine, Army Hospital Research & Referral, Delhi Cantt, New Delhi, India; 2 Indian Naval Hospital Ship, Sandhani, Mumbai India

**Keywords:** Monoallelic, Macroglossia, Neurodevelopmental, Genotype, WAC, *In-silico* analysis

## Abstract

**Background:**

DeSanto-Shinawi Syndrome (DESSH) is a rare neurodevelopmental disorder characterized by intellectual disability, behavioral abnormalities, and distinctive dysmorphic features, linked to likely pathogenic/pathogenic variants in the *WAC* gene. We report the first documented case of DESSH in India, identified in a 3-year-old male presenting with global developmental delay and coarse facies.

**Results:**

Exome sequencing revealed a novel heterozygous nonsense likely pathogenic variant (c.1661 C>A(p.Ser554*)) in the *WAC* gene, expanding the genotypic spectrum associated with this condition. We employed computational methodologies to understand the effects of this novel variant on protein structure and function. In-silico prediction score suggested protein truncation due to the c.1661 C>A (p.Ser554*) variation in the *WAC* gene, expected to result in a loss of normal protein function.

**Conclusion:**

The findings advocate for increased awareness and genetic testing in atypical cases to facilitate accurate diagnosis and management. This case underscores the importance of considering DESSH in the differential diagnosis of similar neurodevelopmental disorders and enhances our understanding of the genetic diversity within the *WAC* gene.

**Supplementary Information:**

The online version contains supplementary material available at 10.1186/s40348-025-00193-1.

## Background

DeSanto-Shinawi Syndrome (DESSH, OMIM # 616708) represents a distinctive neurodevelopmental disorder characterized predominantly by intellectual disability, behavioral issues, and unique facial dysmorphisms. Originally identified through genetic studies that revealed critical likely pathogenic/pathogenic variants in the *WAC* gene, DESSH has since been recognized as a clinically heterogeneous condition with a broad spectrum of phenotypic manifestations [1]. The *WAC* gene, located on chromosome 10p12.1, plays a vital role in neural development and cellular transcription regulation, with its disruption leading to significant developmental consequences [2].

Despite growing awareness, DESSH remains underdiagnosed, primarily due to its phenotypic overlap with other neurodevelopmental disorders such as mucopolysaccharidosis, coffin-siris, and prader-willi syndromes [1]. This overlap often leads to diagnostic challenges, making genetic analysis a crucial component of the diagnostic process. Advances in genomic technologies, particularly exome sequencing (ES), have enhanced our ability to identify novel variants responsible for such syndromes, thereby aiding in more accurate diagnosis and better understanding of these complex disorders [3]. The current report details the first documented case of DESSH in India, marked by a novel likely pathogenic variant in the *WAC* gene. This case not only contributes to the expanding geographic and genetic spectrum of DESSH but also highlights the critical role of tailored genetic investigations in elucidating atypical presentations of known genetic disorders. Through this report, we aim to underscore the importance of considering DESSH in differential diagnosis when faced with variable presentations of developmental delays and dysmorphic features.

## Case presentation

**Fig. 1:**
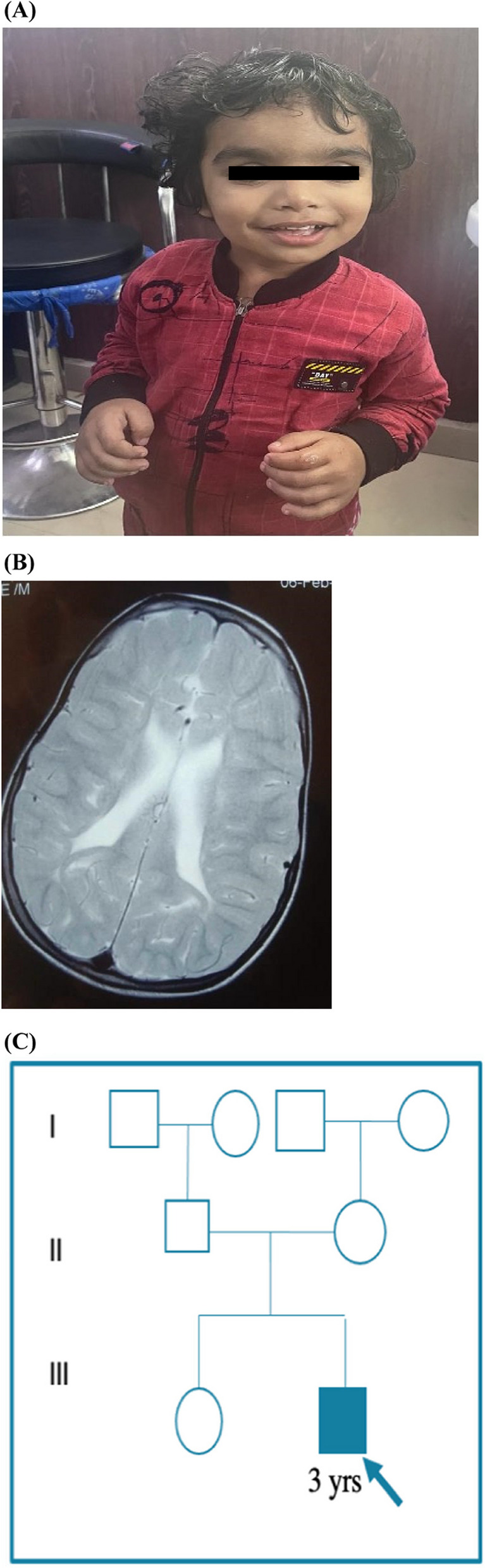
**A** Proband (age 3 years old) with broad forehead, hypertelorism, bushy eyebrows, bulbous nasal tip, flat nasal bridge, macroglossia and coarse facies.** B** T2 weighted axial section of MRI revealed normal myelination for age with mild periventricular leukomalacia at 3.5 years of age. **C** Pedigree of family history

A 3-years-old male, the second child of a non-consanguineous Indian couple (Fig. 1C), was brought with concerns of delay in achieving age-appropriate milestones since early infancy. He was born at 38 weeks via spontaneous vaginal delivery with a birth weight of 3000 grams. Details on birth length and head circumference were not recorded. His prenatal and birth histories were unremarkable, including a negative newborn hearing screening. Detailed developmental history revealed global developmental delay, predominantly affecting language, social & adaptive behaviour. His developmental quotient was 70. There is no history of seizures or any tone abnormality.

During the physical examination, normal anthropometric measurements were noted. Dysmorphic facial features were observed, including a prominent forehead, hypertelorism, bushy eyebrows, a bulbous nasal tip, a flat nasal bridge, macroglossia, and coarse facies, along with mild joint contractures (Fig. 1A). Systemic examination yielded normal results. Initial evaluations, including a complete blood count, thyroid profile, auditory and ophthalmic examinations, were all within normal limits. Mucopolysaccharidosis was kept as differential diagnosis and skeletal survey was performed. It showed mild facial flattening on a lateral skull X-ray, while other images, including those of the dorso-lumbar spine, limbs, chest, pelvis, and hip joints, appeared normal. There were no features suggestive of dysostosis multiplex. Brain MRI revealed periventricular leukomalacia and signs of hypoxic-ischemic encephalopathy **(**Fig. 1B). MPS screening was negative.

## Methods and results

**Fig. 2:**
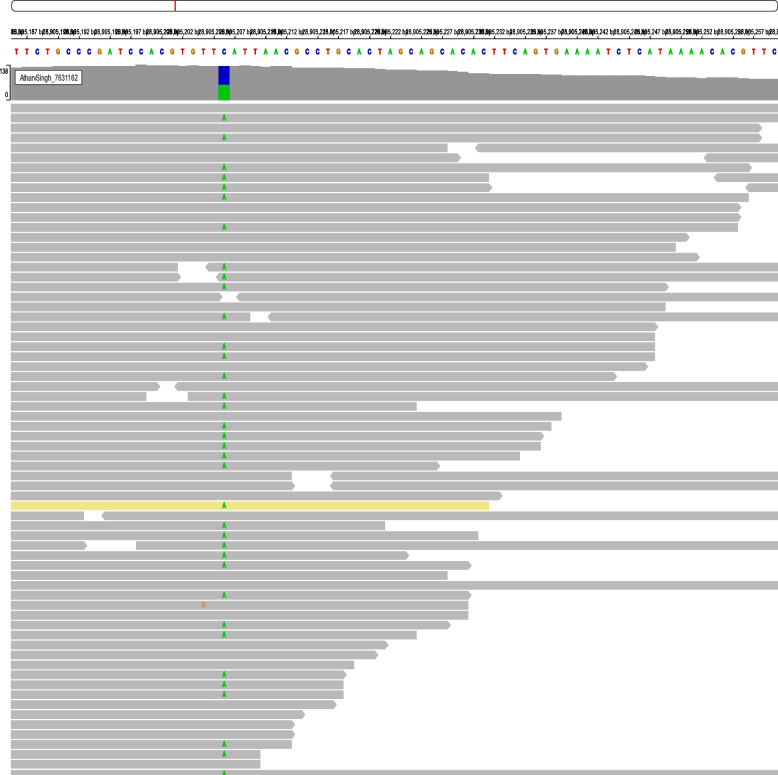
IGV snapshot showing c.1661 C>A variant in *WAC* gene in exome sequencing data

We performed exome sequencing (ES) on DNA obtained from the peripheral blood of the patient and variants found were compared and filtered. Exome sequencing identified a heterozygous nonsense variant c.1661 C>A in the *WAC* gene, predicted to result in a truncated protein (p.Ser554*) (Fig. 2). This variant is classified as ‘likely pathogenic’ by ACMG standards, suggesting a potential loss of function, a common cause of the phenotypes observed in DESSH. Reverse phenotyping confirmed most features were consistent with DESSH. Further segregation analysis of the parents confirmed the variant to be of de novo origin.

### In silico analysis

#### Retrieval of *WAC* protein

The 3D structure of the WW domain-containing adapter protein with a coiled-coil region (WAC) (UniProt ID: Q9BTA9) was obtained from the UniProt protein database [4]. This protein interacts with the RNA polymerase II transcriptional machinery through its WW domain and associates with RNF20-RNF40 via its coiled-coil region. This interaction links and regulates the modification of histone H2BK120ub1 and gene transcription. Additionally, WAC plays a role in regulating cell-cycle checkpoint activation in response to DNA damage [5]. The WAC protein consists of 647 amino acids, as predicted by the AlphaFold tool.

#### Determination of functional effects of nonsense variant

Nonsense variants occur when a premature stop codon is introduced into the DNA sequence. When this mutated sequence is translated into a protein, the resulting protein is incomplete and shorter than normal. As a result, most nonsense variants lead to nonfunctional proteins. We utilized three distinct algorithmic bioinformatics tools—MutationTaster [6], GenoCanyon [7], and FitCons [8]—to evaluate the structural and functional effects of this variant. After being identified as a high-risk nonsynonymous single nucleotide polymorphism (nsSNP) through in silico methods, the variant was further analyzed for its pathogenic score. The analysis revealed that the variant is disease-causing according to MutationTaster, deleterious in the context of GenoCanyon, and harmful as per FitCons (Table 2 in Supplementary material).

#### Prediction study of structural and functional properties of nonsense variant (c.1661 C>A (p.Ser554*))

A web-based application called MutPred v1.2 [9] was used to identify whether amino acid changes in the WAC gene are neutral or associated with disease. MutPred integrates various genetic and molecular data to probabilistically assess the pathogenicity of amino acid substitutions. To use the tool, the WAC protein sequence was input in FASTA format, including its stop gain alterations. The analysis yielded a score of 0.46138, which reflects a general score (g) that indicates the likelihood of the stop gain variant being pathogenic. This score is the average of all neural network scores for loss of function (LOF) from MutPred. The interpreted likelihood score was 0.46138, which is relatively close to 0.50, suggesting a potential pathogenic effect.

#### Analysis of protein structure stability

I-Mutant 2.0 [10] is a platform based on support vector machines that helps predict changes in protein stability resulting from alterations in protein sequences. This tool utilizes data from ProTherm, the largest experimental database of potentially harmful protein variations. The reliability index (RI) for predictions ranges from 0 to 10, with a score of 10 indicating the highest level of reliability. For the WAC protein sequence, the impact of the most damaging nonsynonymous single nucleotide polymorphism (nsSNP) was assessed. The predicted score is ΔΔG = − 1.35 Kcal/mol, which suggests a decrease in protein stability due to the mutation, under constant conditions of 25℃ and a pH of 7.0.

#### Protein evolutionary conservational analysis

**Fig. 3:**
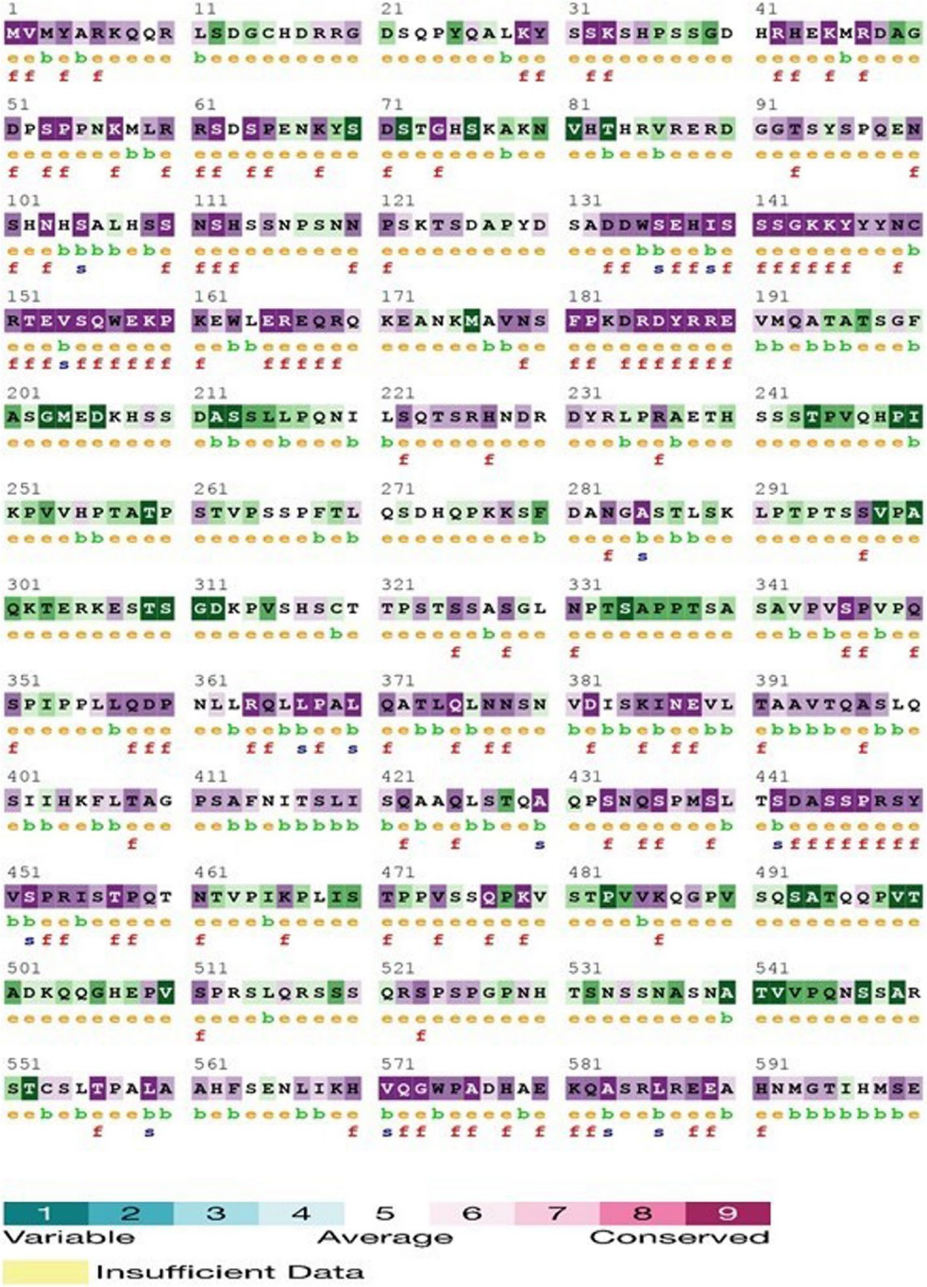
ConSurf results for residues conservation. Colors of ConSurf results showing the level of confidence for the sequence conservation where sky-blue color indicates variables, and dark purple color indicates highly conserved residues

The evolutionary conservation of amino acid positions in protein sequences was evaluated using the ConSurf bioinformatics tool [11]. This study estimates the degree of conservation of amino acid residues based on the evolutionary relationships between homologous sequences. An exposed residue near a high-risk nonsynonymous single nucleotide polymorphism (nsSNP) was selected for further analysis, as shown in Fig. 3. The ConSurf results display the level of confidence regarding sequence conservation using a color gradient from blue to purple. In this scale, blue indicates high variability, while purple signifies highly conserved positions. ConSurf (Fig. 3) also predicts whether the variations are buried (b) or exposed (e), as well as whether they are functional (f) or structural (s) on a scale from 1 to 9. The Consurf predicted amino acid (p.Ser554*) changed with high confidence score (− 0.359) (Table 3 in Supplementary material), which revels exposed residue according to neural network algorithm including their functional and structural consequences.

#### 3D structure analysis

**Fig. 4:**
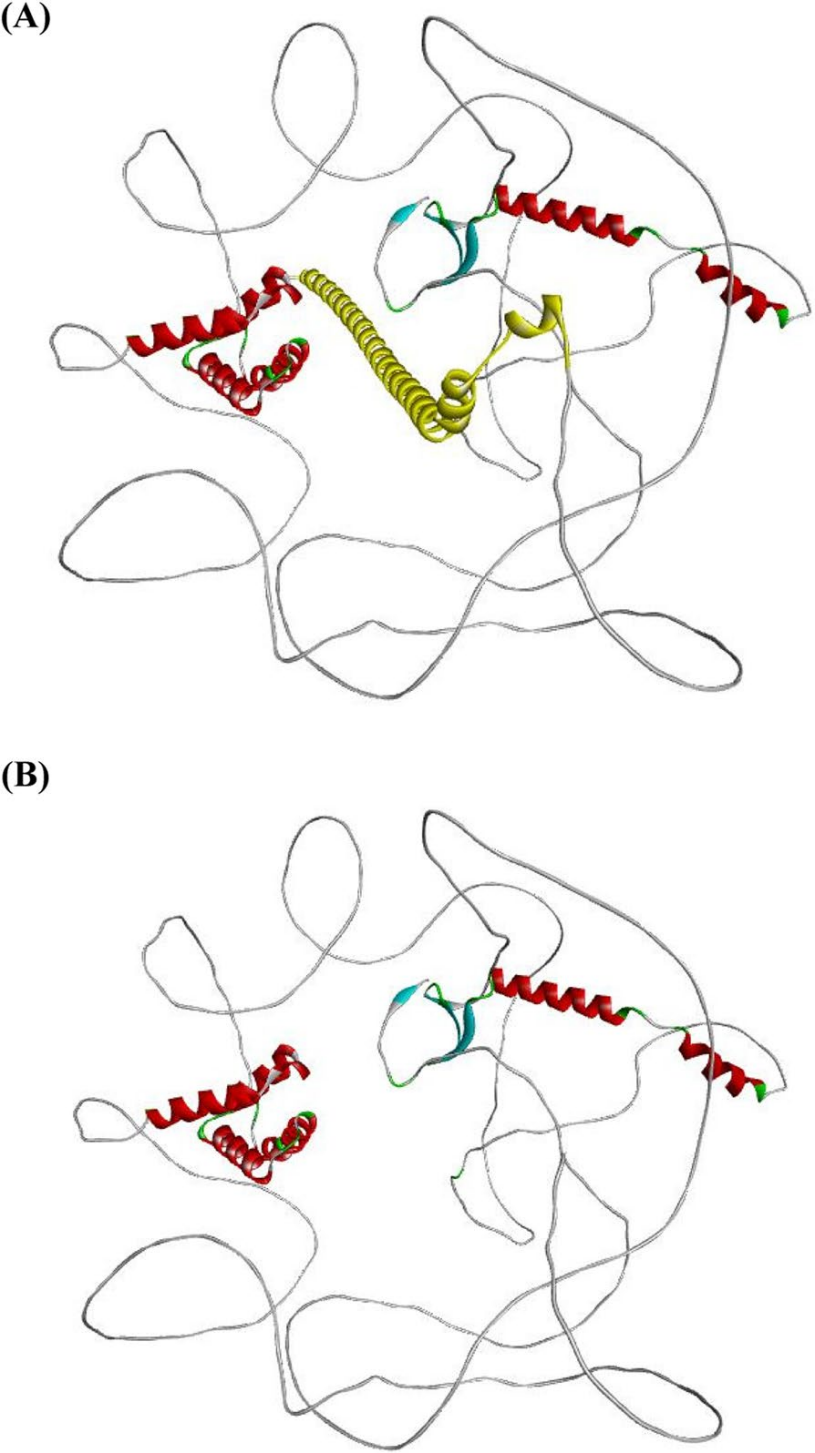
**A** *WAC* 3D structure with 647 amino acids (**B) S**hortened protein structure caused by a nonsense mutation in the *WAC* gene

The structural alterations were visualized using the PyMOL program [12]. Figure 4A shows the normal 3D structure of the WAC protein, which consists of all 647 amino acids. Notably, amino acid 554 (Ser) is highlighted, indicating where a nonsense variant leads to a halt in the translation process. Figure 4B illustrates the shortened protein structure resulting from the nonsense variant in the WAC gene. The c.1661 C>A (p.Ser554*) variant in the WAC gene is predicted to result in a loss of normal protein function due to protein truncation. This stop-gain variation occurs in an exon of the WAC gene, above the region where nonsense-mediated decay is expected to occur.

## Discussion

DeSanto-Shinawi Syndrome (DESSH) is a rare genetic disorder primarily caused by loss-of-function variants in the WAC gene. This condition leads to a variety of clinical manifestations, including developmental and speech delays, behavioral issues, and distinctive dysmorphic features [13]. The WAC gene plays a crucial role in various cellular processes, such as transcription regulation and cell body modulation, which helps explain the complex phenotype observed in patients with DESSH [14].

A novel heterozygous nonsense variant (NM_016628.5; c.1661 C>A) in the WAC gene has been identified, which is associated with the clinical phenotype observed in previously reported cases of DeSanto-Shinawi Syndrome (DESSH) (Table 1) [2]. This likely pathogenic variant results in a truncated protein, which may contribute to the loss of function and manifest as the patient’s symptoms, including dysmorphism, coarse facial features, hypertrichosis, global developmental delay (GDD), and intellectual disability (ID) [15]. Seizures, abnormalities in neuroimaging (ventriculomegaly, cerebral white matter hyperintensities, corpus callosum agenesis etc), cardiac and GI anomalies have been variably reported in patients of DESSH. Review of previously published cases (Table 1) suggests that intellectual disability predominantly language delay is most common feature observed in DESSH (> 90 percent). Hypotonia is present in about 50 percent of the patients. Common dysmorphic features include synophyrs (55%), bulbous nose (50%) and abnormality in palpebral fissures. The variability in phenotypic expression among individuals with DESSH can be attributed to allelic heterogeneity in WAC gene. Additionally, the clinical heterogeneity and severity of DeSanto-Shinawi Syndrome may be influenced by various epigenetic factors, including DNA methylation, histone modifications, non-coding RNAs, and environmental factors.

**Table 1:** Clinical phenotype of our patient compared with those of previously published cases with WAC variants [16]

**Phenotypes**	Current report	Total	Pasquali D et al. [16]	Morales et al. [17]	Alawadhi et al. [18]	Leonardi et al. [19]	Zhang YJ et al. [20]	Vanegas et al. [3]	Lugtenberg et al. [21]	DeSanto et al. [13, 22]	Uehara et al. [23]
**Phenotype present in total cases/Total reported cases**											
Dysmorphic features											
Prominent Forehead	Present	13/36	1/3	0/5	1/4	2/3	0/1	1/1	2/10	6/6	0/3
Hypertelorism	Present	6/36	1/3	0/5	0/4	0/3	0/1	1/1	1/10	3/6	0/3
Synophrys	Present	20/36	3/3	3/5	1/4	2/3	0/1	1/1	3/10	6/6	1/3
Deep set eyes	-	12/36	3/3	0/5	0/4	2/3	0/1	1/1	3/10	3/6	0/3
Palpebral Fissures (down slanting/long)	-	18/36	2/3	0/5	2/4	0/3	0/1	0/1	10/10	1/6	3/3
Bulbous nose	Present	17/36	2/3	1/5	2/4	1/3	1/1	1/1	0/10	6/6	3/3
Depressed nasal bridge	Present	6/36	1/3	0/5	2/4	0/3	0/1	0/1	1/10	2/6	0/3
Depressed philtrum	-	5/36	2/3	0/5	0/4	1/3	0/1	1/1	0/10	1/6	0/3
Macroglossia	Present	1/36	0/3	0/5	0/4	0/3	0/1	0/1	0/10	0/6	1/3
Thin upper lip vermilion	-	7/36	2/3	0/5	1/4	1/3	0/1	0/1	0/10	2/6	1/3
Broad Mouth	Present	14/36	0/3	2/5	0/4	1/3	0/1	0/1	10/10	1/6	0/3
Hirsutism	Present	11/36	2/3	1/5	2/4	1/3	0/1	1/1	1/10	2/6	1/3
Preauricular Pit	-	2/36	0/3	0/5	0/4	0/3	0/1	1/1	0/10	0/6	1/3
Prominent ears	-	1/36	1/3	0/5	0/4	0/3	0/1	0/1	0/10	0/6	0/3
Low set ears	-	5/36	0/3	0/5	1/4	0/3	0/1	1/1	1/10	1/6	1/3
Malar flattening	Present	2/36	1/3	0/5	0/4	0/3	0/1	1/1	0/10	0/6	0/3
Abnormal digit morphology	-	13/36	0/3	2/5	1/4	1/3	0/1	0/1	6/10	0/6	3/3
Developmental problems											
Intellectual disability	Present	27/36	2/3	5/5	2/4	2/3	1/1	1/1	8/10	3/6	3/3
Language delay	Present	33/36	2/3	5/5	3/4	3/3	1/1	1/1	9/10	6/6	3/3
Motor delay	Present	29/36	2/3	5/5	0/4	3/3	1/1	1/1	9/10	6/6	2/3
Behavioral problems											
Autistic features	Present	13/36	1/3	2/5	2/4	2/3	0/1	0/1	4/10	1/6	1/3
ADHD	Present	9/36	0/3	0/5	1/4	0/3	0/1	1/1	4/10	3/6	0/3
Hyperactivity	Present	10/36	3/3	0/5	0/4	1/3	0/1	1/1	4/10	1/6	0/3
Anxiety	-	12/36	3/3	0/5	0/4	1/3	0/1	1/1	3/10	3/6	1/3
Sleep disturbances	-	13/36	3/3	0/5	0/4	2/3	0/1	0/1	6/10	2/6	0/3
Neurological problems											
Hypotonia	-	19/36	1/3	2/5	0/4	2/3	1/1	1/1	6/10	6/6	0/3
Seizures	-	15/36	1/3	5/5	4/4	1/3	1/1	0/1	1/10	2/6	0/3
Other											
Brain abnormalities	Periventricular leukomalacia	8/36	1/3	0/5	2/4	0/3	0/1	0/1	4/10	1/6	0/3
Visual impairment	-	16/36	3/3	1/5	2/4	1/3	0/1	0/1	5/10	4/6	0/3
Hearing impairment	-	4/36	1/3	0/5	0/4	0/3	0/1	1/1	0/10	2/6	0/3
Cardiac abnormalities	-	2/36	2/3	0/5	0/4	0/3	0/1	0/1	0/10	0/6	0/3
GI features	-	13/36	3/3	0/5	0/4	2/3	0/1	1/1	0/10	6/6	1/3

Clinically, DESSH shares features with several other neurodevelopmental disorders, such as mucopolysaccharidosis, Coffin-Siris syndrome, Prader-Willi syndrome, and Smith-Magenis syndrome, which complicates the diagnostic process. Distinguishing DESSH from these conditions is critical, emphasizing the importance of genetic testing in achieving an accurate diagnosis [24].

The discovery of new variants and detailed characterization of individuals with known *WAC* variants enhance our understanding of the genotype-phenotype correlations and expand the genotypic spectrum associated with DESSH. Continued research is essential for refining diagnostic criteria and improving management strategies for this complex disorder, highlighting the need for more comprehensive genetic studies in varied populations.

This case contributes significantly to the growing body of genetic data on DESSH, offering insights that could impact future research and treatment strategies. The identification of a novel variant enriches our genetic databases and helps create a more detailed understanding of the genotype-phenotype relationships associated with this syndrome. Additionally, this case study highlights the considerable overlap in symptoms between DESSH and other similar genetic disorders.

## Conclusions

The identification of a novel *WAC* gene variant in a young patient from India presents a significant addition to the genetic landscape of DeSanto-Shinawi Syndrome (DESSH). This case highlights the critical importance of genetic testing in the differential diagnosis of neurodevelopmental disorders, particularly when clinical presentations are ambiguous and overlap with other syndromes. The novel likely pathogenic (c.1661 C>A) variant discovered through our investigation not only expands our understanding of the genetic underpinnings of DESSH but also serves as a crucial reminder of the diversity within genetic variants contributing to similar clinical phenotypes.

The findings advocate for the integration of comprehensive genetic analysis into clinical practice, ensuring that patients with unexplained neurodevelopmental delays and dysmorphisms receive a timely and accurate diagnosis.

## Supplementary Information

Supplementary Material 1.

## Data Availability

No datasets were generated or analysed during the current study.
