# Supplementary material for: Novel WAC gene variant identified in the first documented case of DeSanto-Shinawi Syndrome in India
Source: Mol Cell Pediatr. 2025 May 10;12:7. doi: 10.1186/s40348-025-00193-1 (PMC12065696; doi:10.1186/s40348-025-00193-1)
Supplement: Supplementary file 1 — Supplementary Material 1. [file 40348_2025_193_MOESM1_ESM.docx]

**Table 2:** *In-silico* prediction score nsSNP

| ***In-silico* functional prediction of *WAC* c.1661C>A (p.Ser554*)** | | |
| --- | --- | --- |
| MutationTaster | 1.00 | Disease causing |
| GenoCanyon | 1.00 | Deleterious |
| fitCons | 0.71 | Deleterious |

**Table 3**: Prediction score of Consurf tool

| **Amino acid changed** | **Consurf score** | **Prediction** |
| --- | --- | --- |
| Ser554* | -0.359 | An exposed residue according to the neural network algorithm. |
